# “Complete Labelling” and Domestic Prosecutions for Crimes Against Humanity

**DOI:** 10.1007/s10609-021-09426-0

**Published:** 2021-11-17

**Authors:** Javier S. Eskauriatza

**Affiliations:** Birmingham Law School, University of Birmingham, Frankland 2108, Edgbaston, Birmingham, B15 2TT UK

## Abstract

Fair labelling is an established principle of criminal justice that scrutinises the way that States use language in labelling criminal defendants and their conduct. I argue that “complete labelling” is a related but separate principle which has not received any explicit attention from commentators. Whereas fair labelling focuses, usually, on the protection of defendant’s rights, the principle of complete labelling explains and justifies whether the labels attached appropriately represent the nature and scale of the wrong done to the community. As a case study, I apply this lens in the context of regional (U.S./Mexican) criminal justice responses to crimes against humanity perpetrated by “drug-cartels” in the context of the Mexican Drug War. Successive administrations in Mexico and the U.S. have tended to charge cartel leaders (and/or their political supporters) with so-called “transnational crimes” (for example, drug-trafficking, money-laundering, bribery/corruption). This is despite the fact that many of the most powerful cartels have controlled territory, attacked entire towns, carried out acts of terror, and disappeared thousands of people. The principle of complete labelling is useful in normative terms because it helps in the critical examination of a State’s prosecutorial practices, exposing problems that might otherwise be missed. In relation to the case study discussed, for example, a focus on complete labelling helps to expose the regional prosecutorial policy as either an unjustified exercise in selectivity or, at worst, an expression of collective denial. After considering certain counteracting reflexions which speak to some of the foundational anxieties of international criminal justice, the article concludes that domestic prosecutions for crimes against humanity in the context of drug-cartels may, sometimes, be justified.

## Introduction

Drug-cartels wreak havoc.^1^ Some are responsible for forced disappearances, mass executions, and spectacular acts of terrorism. They can exercise control over vast swathes of territory. Some capture the legitimate structures of government and exercise a sort of quasi-rule over populations. They kill thousands with impunity. So, when captured and prosecuted, should their leaders answer for crimes against humanity, as well as drug-trafficking, money laundering and bribery? What labels are most appropriate? What labels are fair?

In criminal law, whether domestic or international, the principle of fair labelling helps to scrutinise the messages that different criminal labels carry for a number of different audiences – most obviously, the offender, the victims and survivors, and the broader community that the criminal law purports to regulate.^2^ Labels should be fair, to the defendant, primarily. However, fair labelling does not stop there. Criminal trials should not be understood only as instrumental events that identify guilt, or innocence. They are also communicative processes and, therefore, labels should describe accurately the wrongdoing in question for victims, survivors, and the broader community.^3^ In this respect, incomplete labelling can frustrate the communicative or symbolic function of criminal trials and criminal punishment.^4^

In this article, I explore the concept of labelling in relation to domestic prosecutions for crimes against humanity perpetrated by leaders of powerful drug cartels. I argue that the principle of “complete labelling” helps to reveal and evaluate what may amount to an under-representation of the misconduct of individuals in the context of prosecutorial policy. In Part 1, I begin by tracing the notion of completeness in the fair labelling scholarship of England and Wales and by explaining the relevance of this principle in domestic and international criminal law. In Part 2, I will focus on a case study that permits a practical exploration of the principle of complete labelling. This is an extreme case of criminality that has received an incomplete prosecutorial response, i.e., crimes against humanity perpetrated by the Zeta drug-cartel in North-Eastern Mexico. Although the cartel is responsible for the disappearance of thousands of civilians, the leaders have been charged and convicted only with money-laundering, bribery, drug-trafficking, and firearms offences.^5^ It is undeniable that these labels represent accurately *some* of the wrongdoing involved.^6^ However, there is some discomfort caused by the fact that the more serious crimes, for example, attacks on entire towns, and mass forced disappearances, have remained outside the official regional prosecutorial approach.^7^ How should we understand this prosecutorial selectivity in domestic prosecutions of international crimes? I argue that the principle of complete labelling urges the prosecutors of national criminal justice systems to consider the impact of their selective approach from the perspective of victims, survivors and the wider community affected by the relevant conduct. This is more important in places with endemic levels of corruption and drug-related violence; where the discovery of mass graves has become depressingly common. In Part 3, however, I briefly summarise some important counteracting reflexions which speak to the fundamental anxieties of international criminal justice as a project. In conclusion, I suggest that domestic prosecutions for crimes against humanity could be an important part of a multifaceted approach to tackle the root causes of widespread drug-related violence. However, the existence of the transnational criminal law regime provides an “easy out” for governments keen to avoid the stigma associated with the crimes against humanity label. After all, gross human rights abuses perpetrated against civilians on a massive scale on the national territory implicate the moral responsibility, if not the criminal liability, of the State.

## History of an Idea(l): “Representative”, “Fair”, and “Complete Labelling”

The labels of the criminal law are linked to the moral censure expressed by the operation of the system. Any general theory of criminal labels, therefore, ought to be founded on those principles which best explain and justify the operation of the criminal justice system as a whole.

In the law of England and Wales, the principle of fair labelling was initially understood as being focused on fairness to defendants.^8^ Andrew Ashworth argued that the principle of “representative labelling” meant that “ [t]he label applied to an offence ought *fairly to represent the offender’s wrongdoing*” and that “widely-felt distinctions” between offences ought to be preserved.^9^ Otherwise, “the spirit” of the subjective principle of criminal liability would be violated.^10^ The principle of fair labelling foregrounds any potential future discrimination against defendants by ensuring that the label applied to the defendant does not *over-represent* their wrongdoing (to victims, survivors, and the community). In response to Ashworth’s article, Glanville Williams argued that fairness to defendants was only a part of the picture, arguing that “the notion of fairness can also be made to work the other way: it may be said to be *unfair to the prosecution or to the public if the conviction understates the offender’s fault* [emphasis added].^11^ This is an implicit recognition of the importance of what I refer to here as the principle of complete labelling. Some may argue that complete labelling is really an under-appreciated dimension of the better-known principle of fair labelling. That is fine. However, to avoid the same principle standing as a placeholder for a range of different normative considerations (fairness to whom?), I think it is better to appreciate that complete labelling is really a separate (but, obviously, related) principle of criminal justice.

The principle of complete labelling is founded on the explanation of criminal trials as “public forums” where the defendant, the State, and the victims, are engaged in a communicative process; a process that involves establishing the wrongs done to the community and denouncing them.^12^ According to Joel Feinberg, this communicative process is an opportunity for the State to practice the “authoritative disavowal” or “symbolic nonacquiescence” of the conduct in question.^13^ Whether the defendant’s conduct is labelled as “murder” or “manslaughter”, or “theft” or “robbery”, reflects the outcome of a communicative process that ends with a message sent to the community about the conduct in question.

This process sometimes reveals an uneasy relationship between the search for *the* historical truth of the criminal event and the task of criminal fact-finding.^14^ This is, especially, true when criminal justice is adopted as a response to atrocities.^15^ But a failure to acknowledge the most significant aspect of the defendant’s conduct at all sends a message that the State is indifferent about that behaviour and its consequences – or even worse, the official silence may be interpreted as connivance. Thus, complete labelling is about the effectiveness and legitimacy of the public disavowal involved in a criminal justice process. A lack of accuracy in the portrayal of the wrongdoing at issue may affect the legitimacy of the criminal justice response.

Where the relevant prosecutorial silence amounts to conduct that may rise to crimes against humanity, the stakes, in terms of the legitimacy of the criminal justice response are far higher. As mentioned, widespread human rights abuses implicate the responsibility of the State. Therefore, as a matter of principle, it is even more important for the State to identify accurately, and to disavow, the relevant conduct by the operation of the criminal law. There is a meaning to this public disavowal that goes beyond the immediate consequences of the criminal trial and towards the strengthening of the legitimacy of the State.^16^

Indeed, these considerations can be appreciated in the history of the emergence of the crimes against humanity label itself. Talita Dias has argued that fair labelling was an (implicit) concern in the early development of international crimes.^17^ She argues, rightly, that the terms “genocide” and “crimes against humanity” were proposed as more accurate descriptors of a special kind of offending which concerned the international community as a whole.^18^ As Dias argues, “the definition of ordinary crimes and, in particular, their labels *did not fully capture* the international dimension, the special gravity and the degree of condemnation that were associated with acts or omissions that are now to be international crimes [my emphasis]”.^19^

Certainly, the labels mattered. However, I am less sure that Lemkin, Lauterpacht, or the others, were overly concerned, specifically, with “fairness” in labelling; especially, in the “defendant-rights” dimension that concerned Ashworth, and that now appears in modern textbooks on international criminal law.^20^ Instead, the creation of new crimes to describe especially horrific conduct might better be described as an expression of “completeness” in labelling – an attempt to “convey a more complete and proportionate sense of the criminal wrongdoing at issue”.^21^ Furthermore, there was a sense that these criminal labels did not just describe the past, but they also shaped the future. The emergence of the European human rights movement is owed, at least, in part, to the criminal justice response to Nazi crimes.^22^

The labels of international criminal law are especially condemnatory, but the use of these labels is not determined exclusively by international prosecutions. Indeed, the modern system of international criminal law is established on the principle of complementarity – national courts have the primary responsibility to administer the system of international criminal justice.^23^ This bifurcated system can lead to problems, however, from the perspective of the demands of complete labelling. In particular, as mentioned, crimes against humanity is a label that does not only condemn the behaviour of certain individuals as especially egregious but it also signals the partial breakdown of liberal democratic systems of governance.^24^ Some of the complexity and sensitivity of international criminal justice stems from the fact that criminal proceedings are often putting the State itself on trial (directly, or indirectly).^25^ These tensions between individual and collective wrongdoing at the centre of the international criminal justice project raise countervailing considerations that will be discussed in Part 3. For now, suffice it to say that it is unsurprising that domestic prosecutions for crimes against humanity are still rare and that is, of course, one reason for the emergence of international courts.^26^

The principle of complete labelling has a general application in liberal criminal justice systems. In order to demonstrate its theoretical and practical application, this article is focused on the regional prosecutorial response to crimes perpetrated by drug-cartels as a result of the Mexican Drug War. Despite very high levels of violence and human suffering, this is the type of situation that is usually considered outside of the legal and moral geography of international criminal law.^27^ Therefore, it is a very useful case study for the evaluation of the content, impact, and operation of the principle of complete labelling. In the context of the “war on drugs”, successive Mexican and U.S. governments have been unwilling to attach accurate, complete labels to those implicated in atrocity crimes – international criminal law, as a part of a domestic, or regional, communicative system of denunciation against mass violence and human suffering lays dormant in these jurisdictions.^28^ Instead, the regional response is to investigate and prosecute leaders of powerful and violent organised crime groups for so-called “transnational crimes” (usually, drug-trafficking, bribery, and money-laundering). These labels fit, in part. Some of the wrongdoing is accurately identified and communicated to the communities in question. However, thinking about completeness in criminal labels puts us in mind of the thousands of victims and survivors of Zeta cartel attacks – in this sense, the labels appear to under-represent, at least, *some* of the wrongdoing.

Consider the convictions of the leaders of the Zeta cartel, which forms the basis of the case study in this article. Between 2009–2012, the Zeta cartel was one of the most powerful organisations in the country and it controlled vast swathes of territory (often in collusion with local government). It did not have a political objective other than to control, expand, and defend, lucrative, and illegal, income streams. The leaders were eventually caught, prosecuted, and convicted. Miguel Ángel Treviño (alias “Z-40”) was charged with the possession of an illegal firearm and money-laundering type offences.^29^ He still faces charges in a U.S. indictment, but these extend only to violations of drug-trafficking charges under Title 21 USC Sections 959, 960 and 963.^30^ Omar Treviño, who replaced Miguel as leader of the cartel, was sentenced to 18 years in prison for money-laundering and firearms charges.^31^ Another leader of the Zetas (their nephew) Juan Francisco Treviño, was sentenced to 20 years after being convicted of seven charges of drug-trafficking.^32^ High-ranking political leaders, such as the governors of Coahuila at the material time, have also been investigated and prosecuted. Jorge Torres López, an interim governor during some of the worst violence, was extradited to the U.S. on 29^th^ October 2019, and he pleaded guilty to money-laundering and bribery charges, attracting a 36-month sentence.^33^

In all these cases, no attempt has been made to investigate and prosecute the responsibility of these individuals for crimes against humanity in relation to gross human rights abuses perpetrated by the Zeta cartel upon the citizens of Coahuila (and other regions). Other leaders of transnational organised crime in Mexico have faced investigations and prosecutions but crimes against humanity are absent from the prosecution and conviction of Joaquín Guzman, aka “El Chapo” (in the U.S.), and in the ongoing investigation and prosecution of the ex-Minister for Public Security, Genaro García Luna.^34^ The prosecutorial policy, on both sides of the border, seems to be focused on avoiding those labels that best explain, at least, some of the responsibility in question. The labels used (money-laundering, possession of firearms, and drug-trafficking) may only represent one dimension of the wrongdoing done to the communities by the leaders of organised crime. True, as a matter of fair labelling, it is not, necessarily, unfair to them – a drug cartel can be built around drug-trafficking and the laundering of the proceeds. However, it does not tell the complete story. The “turn to transnational crimes” tends to de-emphasize that part of the story which is founded on the realities of State failure and the breakdown of governance systems. It tends to emphasise that part of the story which tells of a State struggling against the emergence of criminal gangs and lays the blame for the violence on matters beyond State control. The criminal labels, and the regional prosecutorial response, at times appear incomplete.

Given the nature and scale of some of the violence, the most obvious avenue towards a more complete labelling of the wrongdoing involved is to investigate and prosecute cartel leaders for the commission of crimes against humanity when the evidence so demands.^35^ This label captures more fully the scale of some of the wrongdoing as a systematic abuse of human rights perpetrated by a non-State armed group. It also carries important signals to local, national, and international communities about the scale of the problem of the Mexican Drug War (and the related global “war on drugs”). Indeed, it is this message that is occluded by the current approach which focuses on transnational regulatory and financial offences. A focus on the principle of complete labelling may reveal this prosecutorial practice as an exercise in selectivity – a way of ensuring that the war on drugs is not tainted by the morally-loaded labels of international criminal law. In this sense, the principle of complete labelling also contributes to an understanding of the way that States (through their domestic prosecutions) weaponize the fuzzy boundary between so-called “treaty crimes” (drug-trafficking, money-laundering) and those so-called “core crimes” which are directly criminalised under international law (crimes against humanity).^36^ In actual fact, crimes against humanity, perpetrated by State and non-State actors, have been a direct consequence of the region’s war on drugs.

To be clear, not all drug-related violence (in Mexico, or elsewhere) necessarily rises to the level of crimes against humanity. The question explored in this article, however, is founded on the view that the worse of it does cross the threshold into international crimes, and that ignoring this fact as a matter of prosecutorial policy is unjustified. To demonstrate, this article turns to a doctrinal analysis of the law on crimes against humanity. After all, there cannot be an incomplete labelling where the criminal label is unavailable to prosecutors as a matter of legal doctrine.^37^ Mexico signed the Rome Statute on 7 September 2000 and ratification followed on 28^th^ October 2005. Curiously, though, the definition of crimes against humanity in Mexico’s domestic law does not reflect that which is found in the Rome Statute.^38^ There are no contextual elements in the Mexican law. This removes an important legal obstacle to domestic prosecutions.^39^ However, the following analysis applies the more stringent customary international law rules on point, indicating where the Rome Statute law may have diverged from these. After all, the principle of complete labelling is being discussed here as a general principle of international criminal law.

## The Law on Crimes Against Humanity and the Mexican Situation of Violence (Coahuila, 2009–2012)

The historical development of the law on crimes against humanity has been very disorderly and there are still some doubts about its scope.^40^ As a matter of customary international law, crimes against humanity can be identified by the commission of a prohibited act (i.e. murder, torture, rape) in a specific *context*, namely, a “widespread or systematic attack directed against any civilian population”.^41^ In addition to the contextual requirements, any *individual’s* culpability also turns on whether, the prohibited act forms part of the attack on the civilian population and whether the individual had “knowledge” of the attack on civilians. The central purpose of the following analysis is to discover whether the Zetas crimes in North-Eastern Mexico rise to the level of crimes against humanity so that this label was *available to prosecutors* in doctrinal terms. Whether the relevant individuals were or were not guilty can only be determined in a court of law.

The focus, then, is on the contextual elements. It is the contextual threshold (referred to by the Trial Chamber in *Tadić* as “conditions of applicability”) that “elevates crimes that might otherwise fall exclusively under national jurisdiction to crimes of concern to the international community as a whole”.^42^ Previous contextual requirements, such as the “war *nexus*” and the need for a discriminatory intent are no longer required to “cross the threshold” into crimes against humanity.^43^ This is important insofar as there may be legitimate doubts about whether or not the Mexican situation displays the conditions of one (or more) non-international armed conflicts.^44^

The most important element to consider, for present purposes, is the so-called “policy element”, so this will be dealt with first. The first controversy is about whether this is even required as a matter of custom. At the 1998 Rome Conference, disagreements about whether the “widespread or systematic” test should be conjunctive or disjunctive led to the adoption of Article 7(2)(a) Rome Statute.^45^ According to this provision the attack [on a civilian population] must be “*pursuant to or in furtherance of a State or organizational policy to commit such attack* [emphasis added]”.^46^ The inclusion of this element has been very controversial, but it depends on what the policy element means.^47^ If the policy element is required as a matter of customary law, the key question is whether drug-cartels, such as the Zetas cartel, qualify as the *kinds of organisations* that can perpetrate crimes against humanity.

### “State or Organisational Policy”: Do the Zetas Qualify?

Some commentators have argued that the policy element forms no part of the customary law definition.^48^ For example, writing in 2002, Mettraux argued that “this requirement appears to be contradicted by almost all relevant writing on the subject and by the overwhelming state practice”.^49^ A review of the international legislation prior to the Rome Statute reveals that no previous instrument mentions a “State or organisational policy” element.^50^ Therefore, it is worth placing the “re-emergence” of the policy element, as a matter of customary international law, in its historical context.

As Leila Sadat and others have explained, at the Rome Conference, the relatively late addition of the policy element into the definition of “an attack” was “designed to break a deadlock” between States (not as a proposal to change the customary law).^51^ It was included to ensure that random, isolated, unconnected crimes would be excluded from the jurisdiction of the Court.^52^ After the Rome Conference, the International Criminal Tribunal for the Former Yugoslavia (ICTY) dispensed with any policy requirement in the *Kunarac* case*.*^53^ Subsequent case-law at the International Criminal Tribunal for Rwanda (ICTR) followed suit. The Special Court for Sierra Leone (SCSL), whose Statute was drafted after the Rome Conference, also rejected the need for any attack on civilians to be linked to any State or organisational policy. Thus, Mettraux and others have a point – there is a strong argument to the effect that customary law does not require the attack to be pursuant to a “State or organisational policy” as required by Article 7(2)(a) Rome Statute.

On the other hand, some commentators have argued that the policy requirement was rejected erroneously by the *ad-hoc* tribunals.^54^ It is also significant, as Schabas has noted, that at the Rome Conference, only representatives from the Democratic Republic of the Congo and Jamaica *objected* to the (re-)insertion of a policy requirement in the definition of the crime.^55^ The Rome Statute definition is also the only definition of crimes against humanity to be reached after extensive multilateral negotiations. This gives the Rome definition a special weight in terms of the “legitimacy in the development of international law”.^56^ Thus, in my view, it is no longer appropriate to simply disregard the policy requirement out of hand when it comes to the identification of customary international law on point.^57^ Instead, it is better to focus on what the policy requirement might mean.

One sensible approach out of the dilemma has been found by accepting the requirement as a matter of customary law but “interpreting down” the evidentiary threshold.^58^ The policy requirement when accepted by national jurisdictions (and the ICC) must be interpreted in a way that is consistent with the previous authorities, “as a modest threshold that simply excludes random action”.^59^ This means that it is not necessary for the policy to be formally adopted or declared.^60^ Furthermore, its existence as a matter of fact can be satisfied by inference from the way the acts occurred.^61^

In this respect, the Zeta cartel atrocities in Coahuila and environs easily satisfy the standard required of an organisational policy. As recounted by Aguayo and Dayán, during Zeta control over the region, there was a prison in Piedras Negras (just the other side of Eagle Pass, Texas) that was run by the Zeta cartel.^62^ This is not necessarily rare in Mexico where the National Human Rights Commission has found that 71 out of 154 prisons which they visited could be classified as either “self-governed” or “co-governed”.^63^ In the case of Piedras Negras, however, it was used as a regional headquarters by the cartel for a variety of activities. Aguayo and Dayán explain:

This enclave was key for the Zeta organisation because a) it was a safehouse for Zeta leaders that wanted to hide from federal law enforcement, b) they used it to generate income selling drugs, snacks, charging the prisoners rents for the use of the cells, and renting rooms for conjugal visits, c) it offered them a safe and discrete location to install secret compartments in the vehicles that would transport narcotics to the United States, d) it served as a base to recruit *sicarios* [hired assassins], and e) it was a place to temporarily confine those who had been kidnapped and to torture, execute, and disappear bodies.^64^

The systematic nature with which the cartel disappeared their victims has even given rise to a new verb in the Mexican Spanish: *los zacahuileaban* (to make them *zacahuil*, a kind of stew). The Zetas engaged in significant and repeated attacks against migrants and refugees heading to the Texas border. In many cases, kidnapping them, for the purposes of extortion and, in some cases, conducting mass executions (e.g., as in San Fernando).^65^ According to the Mexican National Commission for Human Rights, 9,758 migrants disappeared across a *six-month* timespan in 2009.^66^ In 2013, the Inter-American Commission on Human Rights published a report citing the involvement of criminal organizations such as the Zetas (but also the Gulf Cartel) in the organized kidnapping and disappearance of (mostly) Central American migrants.^67^ The report continues:

if the CNDH’s [National Human Rights Commission] estimates are used as the baseline, there may have been over 18,000 migrants abducted in Mexico in 2009; this means that these crimes could bring in around 50 million dollars a year for the organized crime groups that engage in smuggling migrants, human trafficking, and drug trafficking.^68^

The UN Committee on the Protection of the Rights of Migrant Workers went further and mentioned explicitly the collusion between criminal gangs and state officials in its 2011 report,

The Committee is deeply concerned by the alarming number of cases of kidnapping and extortion of undocumented migrant workers coming up from the southern border and by the acts of torture and cruel, inhuman and degrading treatment, disappearances and killings of these migrants, primarily at the hands of national and international organized crime groups. The Committee is also concerned by allegations that *public authorities participate in these human rights violations, or that they are carried out with the complicity, consent and/or collusion of federal, state and municipal authorities* [emphasis added].^69^

It is sometimes argued that the attacking organisation must be “State-like” before they qualify as capable of committing crimes against humanity. But this interpretation can be challenged as pure Eurocentrism. International criminal law should not simply reflect the European history of the legal frameworks. Today, it is simply not clear what kind of “State” is being imagined when these interpretive arguments are made. As Mégret has argued, in many places “the state is simply not what it was traditionally imagined to be by international (criminal) lawyers, and its ability to inflict harm may pale in comparison to that of armed groups [sic]”.^70^ Indeed, the ICC has acknowledged the same in *Katanga*, when it ruled that “…a policy may be made by groups of persons who govern a specific territory or by any organization with the capability to commit a widespread or systematic attack…”.^71^ Mexico is a vast territory (by European standards). In Mexico, the State’s presence in several of the more violent regions, including in North-Eastern Mexico, is a matter of ongoing (sometimes, violent) negotiation with multiple armed actors.^72^ Rather than imagining the archetypal State and comparing different organisations to that image, other indicators, such as control over territory, control of the movement of persons, informal taxes/extortion, or the mere capacity to attack civilians must be brought to the fore in the interpretation of the kinds of organisations which qualify as part of the customary law definition.^73^

In this respect, the interpretation of “organisational” provided by the majority in the *Kenya* decision should be preferred. The majority’s (non-exhaustive) factor-based approach identified the following as most relevant: (i) a responsible command or established hierarchy, (ii) capacity to carry out the widespread or systematic attack against a civilian population, (iii) control over part of the territory of a State, (iv) having as a primary purpose criminal activities against the civilian population, (v) the articulation of an intention (explicit or implicit) to attack a civilian population, or (vi) being part of a larger group which fulfils some or all of the above-criteria.^74^

The Zetas organisation qualifies as a relevant organisation on these criteria. It was created out of Mexican ex-special forces personnel and, at the relevant time, had a clearly defined hierarchical leadership. The Zetas were heavily armed, and they had a capacity to carry out widespread or systematic attacks against civilians. Furthermore, the organisation exercised control over the territory in question. This is clear when we consider the “revenge attacks” on the northern towns of Coahuila. As Ginger Thompson explains:

…the [U.S.] Drug Enforcement Administration scored an unexpected coup. An agent persuaded a high-level Zetas operative to hand over the trackable cellphone identification numbers for two of the cartel’s most wanted kingpins, Miguel Ángel Treviño and his ​brother Omar. Then the DEA took a gamble. It shared the intelligence with a Mexican federal police unit that has long had problems with leaks — even though its members had been trained and vetted by the DEA. Almost immediately, the Treviños learned they’d been betrayed.^75^

In response to the betrayal, the Zeta leadership unleashed a wave of attacks on several towns of Northern Coahuila. Although the details are still unclear (owing to a lack of a full investigation) Piedras Negras and Allende were particularly affected with thousands of emergency calls reporting fires breaking out all over the towns.^76^

In particular, the facts of what happened in Allende are illustrative of what happens when an area is handed over to an organisation like the Zetas drug cartel.^77^ As explained by the investigative director, José Juan Morales, Coahuila State Prosecutor’s Office:

We have testimony from people who say they participated in the crime. They described some 50 trucks arriving in Allende, carrying people connected to the cartel. They broke into houses, they looted them and burned them. Afterward, they kidnapped the people who lived in those houses and took them to a ranch just outside of Allende. First, they killed them. They put them inside a storage shed filled with hay. They doused them with fuel and lit them on fire, feeding the flames for hours and hours.^78^

In relation to the official response, Allende’s deputy mayor has said that “there was a two-sided government, the official one and the criminal one that was in charge. We knew that the police were controlled by criminals”.^79^ To summarise, it may well be that a policy element is now required as a matter of customary international law. However, the threshold is low, and it would be easily satisfied in respect of Zeta cartel crimes. Therefore, the most important contextual element for the purposes of applying the crimes against humanity label to some of Mexico’s drug-related violence is satisfied.

### The Requirement of a “Widespread or Systematic Attack”

For the label of crimes against humanity to apply, there must also be an “attack” on a civilian population. The attack is “the event” during which one of the prohibited crimes must take place. However, it need not be a military attack.^80^ An “attack” is a “course of conduct involving the commission of acts of violence”.^81^ Any individual need only be implicated in a *single* act (i.e., kill one person, or help to imprison them, or deport them) but this prohibited act must form part of the broader attack on civilians.^82^

The Zeta revenge attacks on the northern towns of Coahuila were military-style attacks, as such. Therefore, this aspect of the definition is easily satisfied. It bears mentioning that, as a matter of customary international law, the mistreatment of a civilian population also qualifies as “an attack”. In *Akayesu*, the Trial Chamber held that “*exerting pressure on the population to act in a particular manner*, may come under the purview of an attack, if orchestrated on a massive scale or in a systematic manner [emphasis added]”.^83^ As reported by Aguayo and Dayán, the Zeta cartel charged non-cartel inmates for use of their cells in Piedras Negras prison. They also “forced” inmates to carry out a number of cartel-related activities, including modifying vehicles and participating in the burning of the victims’ bodies.^84^ In relation to the attack on Allende, the population was directed to ignore the attacks on the town. Public service personnel, summoned by the burning of the bodies in nearby ranches, have explained that upon their arrival, they met the Zetas gunmen who told them to withdraw:

They said there were going to be numerous incidents. We were going to get numerous emergency calls about gunshots, fires and things like that. They told us we were not authorized to respond. In my capacity as fire chief, what I did was to advise my boss, who in this case was the mayor. I told him that we were facing an impossible situation and that the only thing we could do was to stand down, out of fear of the threats we faced. There were too many armed men. We were afraid for our lives. We couldn’t fight bullets with water.^85^

There is clear evidence that the Zetas cartel perpetrated attacks on numerous towns in the territory controlled by the organisation. But in addition, the law requires that the attack must be *either* widespread *or* systematic.^86^ As the Appeals Chamber put it in *Tadić,* “the acts of the accused must comprise *part of a pattern of widespread or systematic crimes directed against a civilian population* [emphasis added]”.^87^ In *Tadić*, the Trial Chamber thought that the standard for “widespread” was satisfied by evidence of the “large-scale nature of the attack and the number of victims”.^88^ The ICTR may have embraced a slightly higher threshold in *Akayesu* where the Trial Chamber defined “widespread” as “massive, frequent, large scale action, carried out collectively with considerable seriousness and directed against a multiplicity of victims”.^89^ The ICC jurisprudence has not deviated materially from this customary law understanding of “widespread”.^90^

It is not possible to be absolutely clear about how many victims are needed for there to be a “multiplicity” of victims (beyond the exclusion of isolated acts of violence).^91^ However, the revenge attacks, which, at least, killed hundreds, must be appreciated in the context of repeated crimes against civilians by the Zetas across the North-Eastern territory during the three-year period, including the kidnapping and disappearance of thousands of migrants, and the systematic torture and disappearances at Piedras Negras prison. The precise scope of application of the legal terms cannot be separated from the factual circumstances in question. Thus, a reflexive approach might be wise.^92^ As Jalloh has argued, “the focus of the analysis should […] hone in on the wrongful conduct that has caused international social harm or alarm [*sic*]”, rather than, exclusively, on the meaning of particular words.^93^ This is helpful. The alarm in question is that thousands of persons in North-Eastern Mexico disappeared and mass graves are being discovered with alarming frequency. Many of these are victims of Zeta attacks (though owing to lack of investigations by the State it is impossible to say, at this stage, how many).^94^

An excessively narrow theory of the situation may consider that the Zetas attack(s) are not “widespread”. Even so, they may be considered “systematic”. The jurisprudence of the *ad-hoc* tribunals has equated this criterion with evidence of planning, organisation, and the existence of a regular pattern of conduct. For example, in *Akayesu*, the Trial Chamber defined “systematic” as “thoroughly organised and following a regular pattern on the basis of a common policy involving substantial public *or private resources* [emphasis added]”.^95^ In *Kunarac,* the Appeals Chamber agreed with the Trial Chamber’s view that “patterns of crimes – that is the non-accidental repetition of similar criminal conduct on a regular basis – are a common expression of such systematic occurrence”.^96^ Again, the Rome Statute jurisprudence has followed a similar line. In *Katanga*, a Pre-Trial Chamber stated that “systematic” meant the Prosecutor needed evidence of “an organised plan in furtherance of a common policy, which follows from a regular pattern and results in a continuous commission of acts”.^97^ Alternatively, the Pre-Trial Chamber required evidence of “non-accidental repetition of similar criminal conduct on a regular basis”.^98^

The revenge attacks were certainly systematic. The *Zetas* arrived in town with a purpose and set to the destruction of property and the rounding up of persons associated that they believed were associated with the DEA informant. Violent revenge is by definition non-accidental. Further, the repeated assaults on migrants can also be described as one systematic attack – repeated kidnappings and disappearances are not random events because an income-stream, linked to the repetition of human rights abuses, by definition cannot be random or isolated. Finally, it is not possible to run a prison as a regional headquarters, and site of extermination, without also satisfying the systematic standard.

### “Directed Against Any Civilian Population”

The crimes against humanity label requires that it is a “civilian population” that is protected from widespread or systematic attack.^99^ This, again, suggests that evidence of a certain amount of scale is required in terms of the victims of the attack. It also serves to exclude isolated acts against, even multiple, individuals. But it is clear that customary law adopts a wide definition of a “civilian population”. This is evidenced by the use of the word “any” which means that the distinguishing characteristics of the civilian population are immaterial (i.e. their race, their ethnicity, their nationality).^100^ Problematically, the definition of a “civilian” population tends to suggest a normative coupling with international humanitarian law.^101^ But as Ambos has argued, the two bodies of law are supported by different rationales – namely, the prohibition of crimes against humanity does not arise from the “principle of distinction” and so automatic transfers from one body of law to the other should be avoided.^102^ In peacetime situations, such as (ostensibly) North-Eastern Mexico between 2009–2012, the core of crimes against humanity should be understood as the protection of *everyone* from organised and systematic attacks, regardless of their status, and regardless of the kind of organisation that is carrying out the widespread or systematic attack. This interpretation is most faithful to the rationale of crimes against humanity as protecting the human rights of individuals from organised attack by groups in power; groups that control the territory in which they happen to live, or need to pass through, as a matter of simple chance. As has been explained, the Zetas organisation was in control of vast swathes of Coahuila and colluded with local government to carry out their activities, in Piedras Negras prison, and across the territory, attacking towns and kidnapping and disappearing migrants.

Yet, another issue that might arise is the question of *how many* civilians must be targeted before the “population” criterion is satisfied. The Trial Chamber in *Tadić* stated that the reference to “population” meant that the crimes must have been “crimes of a collective nature” and this, therefore, excluded “single or isolated acts” which might be ordinary crimes or war crimes.^103^ This does not mean, however, that the *entire* population living in the sphere of the attack must have been subjected to the attack.^104^ During the material time, some parts of North-Eastern Mexico may even have been relatively peaceful. Also, in the context of the revenge attacks, the Zetas selected certain families and individuals and spared others. In the prison, there was an aspect of selection in the context of the killings, and in the selection of migrants to kidnap, extort, and disappear. According to witness testimony, for example, the victims in the prison were often drug dealers that were in competition with the Zetas (so-called “grasshoppers”), or people who owed the cartel money, and the family members of these persons. The so-called “kitchens” were also used to disappear people who were taken from the towns of Allende, Morelos, Nava, Villa Unión and Zaragoza during the revenge attacks of March 2011.

Yet, an aspect of selection of victims is not determinative. The victims and survivors were not randomly selected individuals – the killing and burning of civilians was a part of how the Zetas (and other cartels) *governed* in Coahuila. The fact that other cartels operate on a similar basis explains why there are approximately 90,000+ official missing persons in Mexico.^105^ It does not matter that the civilians were only temporarily on the territory in question (i.e., migrants); nor would it matter that the victims were police or local law enforcement.^106^ Even the presence of so-called “self-defence groups” (*autodefensas*) would not deprive the population attacked of its civilian character. The law states that it is sufficient if an organisation attacks *a part* of the population in the area subjected to attack. The Appeals Chamber in *Kunarac* determined that it was:

sufficient to show that enough individuals were targeted in the course of the attack, or that they were targeted in such a way as to satisfy the Chamber that the attack was in fact directed against a civilian population, rather than against a limited and randomly selected number of individuals.^107^

This approach has been endorsed by recent ICC jurisprudence.^108^ It has also been followed in the 2019 Draft Convention on Crimes against Humanity prepared by the International Law Commission which states that population simply means that the attack must be directed against “multiple victims”.^109^ None of the Zetas victims were “randomly selected” – for example, in the context of the revenge attacks, they were aiming at those individuals and families that they decided were linked to the informant. It is true that innocent persons were harmed as a consequence of the application of the policy, seemingly, at random – however, the policy was not to attack random people. Neither is the policy to kidnap, extort, and disappear migrants, random, for the purposes of the law.

Lastly, there is a question about what is meant by “directed against” (or the notion of an attack being “on” civilians). At the ICTY, the “war *nexus*” in the Statute meant that it was necessary to exclude civilians that were “collateral damage”. The Appeals Chamber in *Kunarac* explained that “directed against” signals that the civilian population is the “primary object of the attack”, thus excluding *incidental* harm to civilians.^110^ It found evidence for “direction” from a range of factors including: the means and methods used to attack the victims; their status and number; and the nature of the crimes committed during the attack.^111^ Recent jurisprudence at the ICC has reflected the approach of the *ad-hoc* tribunals. In *Bemba,* the Trial Chamber determined that it was necessary to prove that “the civilian population was the primary, as opposed to incidental, target of the attack…”.^112^ This finding was evidenced by the “means and methods used in the course of the attack, the status of the victims, their number, the discriminatory nature of the attack, the nature of the crimes committed in its course, [and] the form of resistance to the assailants at the time of the attack,[…] ”^113^ In relation to the activities of the Zetas in Coahuila, the “kitchens” of Piedras Negras, the extermination of migrants in San Fernando, and elsewhere, this is not really in doubt.

## Complete Labelling And Countervailing Considerations

Given the nature and scale of the Zeta crimes in Coahuila, and surroundings, I have argued in Part 2 that the most complete and appropriate label for these crimes is crimes against humanity. Similarly, national and international civil society actors have expressed inconformity with the current approach by turning to the language of international criminal law to describe the situation of violence and the responsibility of those involved (including the State). National and international human rights organisations have produced a number of Rome Statute Article 15 communications in recent years alleging that crimes against humanity have been committed by State and non-State actors on Mexican territory in Baja California, Coahuila, and Chihuahua.^114^ In Coahuila, specifically, they have described and evaluated the violence in terms of crimes against humanity as a more appropriate label that tells a more complete story about what happened when the Zeta cartel governed large swathes of Coahuila.^115^ However, there are important counterarguments to consider and this section looks at some of these. The worry is that the prosecution and conviction of drug-cartel leaders for crimes against humanity is, in a different way, *also* failing to adhere to the principle of complete labelling.

In the first place, to avoid selectivity claims, domestic prosecutions for international crimes would seem to require a more serious investigation of the Mexican Armed Forces and their conduct during the Mexican Drug War. Successive governments have been unwilling to hold the military to account.^116^ Thus, any benefits of the criminal trial as a more complete communicative process would appear to be available only to those who happen to be victims and survivors of cartel violence.^117^ This is, clearly, not justifiable, as a matter of principle.

Secondly, questions may be raised about the expressive or symbolic power of domestic criminal trials in Mexico *tout court*. For example, it may be asked how prosecuting cartel leaders for crimes against humanity would adequately express and denounce the collusion between the cartels and State officials that has led to the overall situation of widespread violence and bloodshed. As Chayes has argued, “ [i]n…Mexico […] among other acutely corrupt countries, public officials have entered into destabilizing alliances, even symbiosis, with transnational criminal superpowers: drug and weapons syndicates whose activities span continents”.^118^ In this context, what good is it to insist upon the communicative benefits of domestic prosecutions? Criminal trials are likely to be counter-productive or weaponised when domestic criminal justice systems are compromised. Of course, it may be countered that this is the very reason for the emergence of international criminal courts. Indeed, some commentators have argued that the modern law of crimes against humanity means that the ICC should begin to consider Mexico-type situations more seriously. As Arriaza and Martínez have argued,

Increasingly, situations involving crimes against humanity are likely to involve a murky mix of actions to control territory and resources for personal, organizational or political gain, combined with ever-more sophisticated international networks to fund these actions and hide the proceeds […] For the ICC, and international justice more generally, continued relevance will increasingly require grappling with this underlying reality.^119^

The recent opening of an investigation in the Republic of the Philippines has, at least, put official violence linked to the “war on drugs” on the international criminal justice agenda.^120^ Yet, whether or not the ongoing “pre-preliminary” examination of Mexico leads anywhere is uncertain.

Thirdly, some may argue that to apply the supposedly universal standards of Anglo-American criminal law theory to a radically different context is at best wrongheaded, or at worst, an exercise in a kind of “academic imperialism”. Fair labelling and complete labelling may be relevant to questions of “murder” and “manslaughter” at the “Old Bailey”, but this theory of criminal law is simply not transferable to a prosecutorial policy that involves transnational organised crime and the “war on drugs” in Mexico. To be sure, Feinberg, Duff, and others were simply not thinking about the communicative power of criminal trials in this kind of context. This is a serious charge. As Martii Koskenniemi has argued, when actors refer to the “special character” of certain norms this “enables them to transgress the preferences of single individuals, clans or nations”.^121^ Because reason (in contrast to State will or State consensus) is presented as universal, these commands are presented as enjoying universal validity. Of course, this approach to the identification of the law has a historical resonance in the “civilization” of the peoples of North and South America.^122^

At the same time, the principle of fair labelling is reflected in core texts of international human rights law, and it may be said to form part of customary international law.^123^ My argument is that complete labelling is an under-appreciated dimension of fair labelling which has a distinctive normative pull. In my view, it is better to distinguish complete labelling as a separate principle. It may not have been referred to explicitly in the traditional sources of international law, but I argue that its validity as a general principle can be inferred from the general acceptance of fair labelling. Mexico has accepted the principle of fair labelling insofar as it acceded to the International Convention on Civil and Political Rights.^124^ This provides the necessary consent to the principle of complete labelling. As Gardner explains,

…a norm is valid as a norm of that system in virtue of the fact that at some relevant time and place some relevant agent or agents announced it, practiced it, invoked it, enforced it, endorsed it, or otherwise engaged with it.^125^

Mexico is also a part of the Rome Statute system, and the ICC has referred to the principle of fair labelling in its jurisprudence.^126^

Fourthly, and from a slightly different critical perspective, there is the question of whether the individualisation of guilt sits comfortably with the collective nature of widespread violence in Mexico. It may be an excessively radical liberalism that wants to hold a few individuals to account for what is, in essence, the breakdown of State governance in several places in Mexico.^127^ The flipside of individualisation could also function as a sort of collective amnesty for those elements of the State that have contributed to the breakdown in public security and mass violence in Mexico.^128^ Therefore, perhaps, a more collective public reckoning is required.^129^ The principle of complete labelling may require a synergistic approach – one that attempts to fuse individual and collective accountability.

In an effort to acknowledge the seriousness of the situation, national and international civil society organisations have argued that the current state of affairs is so beyond the pale, that some form of transitional justice mechanism must be established to aid the failing Mexican criminal justice system.^130^ The “Citizen’s Proposal” is one attempt to delineate a transitional justice roadmap that would go some way towards a more complete labelling of the criminality that arises from the horrors of the war on drugs. Among other things, it provides for an international mechanism for the investigation of atrocities in Mexico (*Mecanismo Internacional Contra la Impunidad en Mexico*, known as “MICIM”) and it also sets out plans for a parallel truth commission and reparations programme. As ever, comparisons and lessons might be learned from other “models”, such as, the “Integral System for Truth, Justice, Reparation and Non-Repetition” in Colombia.^131^ The Colombian system includes a number of separate but “integrated” mechanisms including i) a Truth, Reconciliation and Non-Repetition Commission, ii) a Special Search Unit for Conflict-Related Disappeared Persons, iii) the Special Jurisdiction for Peace and iv) a range of specific measures on reparations.^132^

But Mexico is not Colombia – in particular, the most powerful cartels (Cartel de Jalisco Nueva Generación, Cartel de Sinaloa) are not in the same position as the FARC-EP in 2016.^133^ The homicide rate across Mexico remains at an all-time high. A cursory look at the official government figures tells the story of approximately 350,000 drug-related deaths since the war on drugs was declared by ex-President Felipe Calderón in 2006.^134^ Torture continues to be widely practiced by State officials throughout the criminal justice process, including during so-called precautionary detention without charge (*arraigo*).^135^ The femicide rate has increased by 145% since 2015.^136^ Even the Covid-19 pandemic failed to make a dent in the stories of violence and bloodshed.^137^ As is well-known, the general “impunity rate” across Mexico is over 90% and access to justice is, in practical terms, non-existent.^138^

Despite the situation, however, the current Mexican administration (led by President Andrés Manuel López Obrador, hereafter, “AMLO”) has not been amenable to calls for the implementation of a Mexican transitional justice programme. AMLO came to power in late 2018 with the promise to put an end to what he referred to as the “war on the cartels” – under the now much-ridiculed slogan: “hugs, not bullets”.^139^ At first, during the campaign trail, it appeared that this meant that a transitional justice approach was within the realms of the possible. During one of his daily televised morning press conferences, AMLO announced that:

[t]he main function of the government is to guarantee public security, and it is not the strategy of the security forces to detain drug kingpins. What we are seeking is security, that we may diminish the number of daily homicides.’^140^

When asked directly whether his administration would end the “war on the cartels”, he responded: “There is no war, officially, there is no more war. We want peace, and we are going to get peace”.^141^ However, AMLO has backtracked. Mexican armed forces (together with the newly created National Guard) will continue to carry out crucial public security tasks through to the end of his administration 2024.^142^ As such, the war on drugs has continued, along with the concomitant human loss, and the lack of any serious attempt to acknowledge what is happening.

## Conclusion

To quote the Mexican writer, Juan Rulfo: “*No decimos lo que pensamos. Hace ya tiempo que se nos acabaron las ganas de hablar*”.^143^ It may be countered that the crimes against humanity label “covers only a small part of the crime committed” in Mexico as a whole.^144^ As such, the argument goes, the label is not really necessary, it is inaccurate, or perhaps, it unfairly places the consequences of a more collective social breakdown on the shoulders of a few individuals.

This may be true in some cases. Even so, Wirken and Bosdriesz are mistaken in thinking that the elevation of some of the worst violence to the level of crimes against humanity would be “artificial”.^145^ This under-appreciates the value in conceptualizing the worst atrocities in a particular situation as international crimes and the jurisgenerative power of labels in helping to support and implement the most effective policy solutions. At the same time, it may underestimate the damage done by current policy approaches which underplay the levels of violence by ensuring that those individuals who are most responsible are either i) not investigated or ii) convicted of transnational crimes, such as money-laundering and bribery. In terms of criminal justice as communication of moral wrongs, the acknowledgment that international crimes are taking place in, and being produced by the “war on drugs”, could, at least, help to “move the needle” in States like Mexico, closer towards more significant discussions about what should be done with civil society actors and external partners. The Mexican President has been engaged in producing a supposed “4^th^ Transformation” of Mexico.^146^ But this is mere rhetoric, or jargon, unless his government (and those that follow) are willing to accept the nature and scale of the public security problems which show no signs of going away. One strategy could be to pursue domestic prosecutions for crimes against humanity, when the evidence demands it, in order that Mexico can more effectively disavow the relevant conduct, uphold victims’ rights, and create space and support for more meaningful policy responses that tackle the root causes of the Mexican Drug War. Further, from a moral perspective, a failure to condemn international crimes may not be unrelated to a certain connivance in their commission.

The incomplete labelling of defendants has tended to portray the Mexican Drug War problem in a half-light, or even worse, as narco-entertainment. In October 2015, to the profound embarrassment of Mexican authorities, Kate del Castillo (an LA-based Mexican TV and movie star) and Sean Penn (a Hollywood A-lister) met with Joaquín “El Chapo” Guzmán in Mexico to carry out an interview for Rolling Stone magazine and to discuss plans for a biopic – all the time while El Chapo was a leader of one of the most violent criminal groups in the world.^147^ The glamourisation of *narco* culture is part of the problem.^148^

This is not to say that labelling cartel leaders for crimes against humanity will eliminate, as if by magic, the perpetration of widespread human rights abuses in the context of the “war on drugs”. However, at least, in terms of the ongoing fight against “grand corruption”, indictments that equate the criminality of the most powerful drug-cartels with that of other violent non-State armed groups (e.g., Islamic State/Al-Qaeda) may at least illuminate the impacts of kleptocracy, and help to generate the political will towards implementing, *inter alia*, the Citizens Proposal.

This article is founded on the view that international criminal law should be understood as a positive dimension of a broader system of transitional justice – part of the toolkit.^149^ This conclusion may be criticised as an exercise in what Gerry Simpson has called “incrementalism”.^150^ It may also be seen as idealist, as if the critiques, in fact, run deeper and have more problematic implications for the international criminal justice project. However, again, to quote Simpson, the great strength of the criminal justice project, international and domestic, is that most people know that “bad individuals should be jailed in the name of justice and […] it would be unconscionable to remain passive in the face of this”.^151^ As part of this project, I have tried to describe and evaluate the general principle of complete labelling which responds to the requirement that criminal prosecutions represent appropriately the wrongs done by individuals to the broader community. It is related to the principle of fair labelling, but it is less focused on the defendant’s fair trial rights. In fact, it may pull in a different direction, one that is more focused on the victims of crimes and on the future of a broken society. In some instances, this may cause tension where fairness to the community may be unfair to defendants who are identified unreasonably as the bearers of what should be a more collective guilt. At the same time, the individualisation of guilt allows for criminal processes to pursue the aims of retribution and deterrence more effectively.^152^ Finally, as a general principle, complete labelling may inform non-prosecutorial approaches to accountability, i.e., as a foundational norm of transitional justice programmes. Ultimately, if the project of (international) criminal justice is to have any legitimacy, it must be to play a constructive role in helping societies to tell more complete and accurate stories about mass suffering in the hope that stronger communities will emerge. Hope, in this sense, is about commitment. Sometimes, that commitment must be expressed via domestic prosecutions of international crimes.

## Footnotes

1 I use “drug-cartel” to mean non-State armed groups that engage in a range of illicit activities including, but not limited to, the trafficking of illegal drugs.

2 James Chalmers and Fiona Leverick, “Fair Labelling in Criminal Law”, *Modern Law Review* (2008) 217–246, 236; Talita de Souza Dias, “Recharacterisation of Crimes and the Principles of Fair Labelling in International Criminal Law”, *International Criminal Law Review* (2018) 788–821.

3 Anthony Duff, *Answering for Crime* (Oxford, Hart Publishing: 2007); Anthony Duff, “Authority and Responsibility in International Criminal Law”, in Samantha Besson and John Tasoulias (Eds.) *The Philosophy of International Law* (Oxford, OUP: 2010) 589–604, 594.

4 Joel Feinberg, *Doing and Deserving: Essays in the Theory of Responsibility* (Princeton, New Jersey, Princeton University Press: 1970); H.L.A. Hart, *Punishment and Responsibility* (Oxford, OUP: 1968) 6; Anthony Duff, “Authority and Responsibility in International Criminal Law”, *ibid*.

5 It is also true that Mexican Armed Forces have been implicated in gross human rights violations rising to the level of crimes against humanity, however, this is not the focus of this paper. See Open Justice Initiative, *Undeniable Atrocities – Confronting Crimes Against Humanity in Mexico*, (New York, 2006). For a general introduction to the situation of violence in Mexico see Lydia Cacho, Sergio González Rodríguez, Anabel Hernández, Diego Enrique Osorno, Emiliano Ruiz Parra, Marcela Turati, and Juan Villorio, *The Sorrows of Mexico* – *An Indictment of their Country’s Failings by Seven Exceptional Writers*, (MacLehose Press, London: 2016).

6 I do not argue that so-called transnational crimes are “less serious” at a conceptual level, although, there is a difference between some of the regulatory and financial offences prohibited in the “treaty crimes” regime and gross human rights abuses rising to crimes against humanity, see Douglas Guilfoyle, “Transnational Crimes” in Kevin Jon Heller, Frédéric Mégret, Sarah MH Nouwen, Jens David Ohlin, and Darryl Robinson, (Eds.) *The Oxford Handbook of International Criminal Law* (Oxford, OUP: 2020) 791.

7 By “regional criminal justice” I simply refer to the investigation, prosecution, and conviction of cartel leaders by U.S. and Mexican criminal justice systems.

8 Andrew Ashworth, “The Elasticity of Mens Rea” in C. F. H. Tapper (ed.) *Crime, Proof and Punishment: Essays in Memory of Sir Rupert Cross* (London, Butterworth: 1981) 45. It was Glanville Williams that recommended swapping the adjective “representative” with “fair”, see Glanville Williams, “Convictions and Fair Labelling” *Cambridge Law Journal* (1983) 85–95, 85.

9 Ashworth, *ibid*., 53, 54.

10 For a general critique of the focus on this “subjective principle” see Alan Norrie, *Crime, Reason, and History* – *A Critical Introduction to Criminal Law*, 3^rd^ Ed., (Cambridge, Cambridge University Press: 2014).

11 Glanville Williams, “Convictions and Fair Labelling” *Cambridge Law Journal* (1983) 85–95, 85; see also Andrew P. Simester, John R. Spencer, G.R. Sullivan, and Graham J. Virgo, *Simester and Sullivan’s Criminal Law – Theory and Doctrine* (Hart Publishing, Oxford: 2013) 31–32.

12 Anthony Duff, “Authority and Responsibility” (n. 3) 594.

13 Joel Feinberg (n. 4) 101–102.

14 Martti Koskenniemi, “Between Impunity and Show Trials” *Max Planck Yearbook of United Nations Law* (2002) 1–35, 11.

15 Hannah Arendt, *Eichmann in Jerusalem*, (Penguin Books, London: 2006) 253; Robert Cryer, Darryl Robinson and Sergey Vasiliev, *International Criminal Law and Procedure*, 4^th^ Ed. (Cambridge, CUP: 2019) 38–42.

16 Barrie Sander, “The Expressive Turn of International Criminal Justice: A Field in Search of Meaning” *Leiden Journal of International Law* (2019) 851–872, 852.

17 Dias, (n. 2) 789. See also David Nersessian, “Comparative Approaches to Punishing Hate: The Intersection of Genocide and Crimes Against Humanity”, *Stanford Journal of International Law* (2007) 221–264; Douglas Guilfoyle, “Responsibility for Collective Atrocities: Fair Labelling and Approaches to Commission in International Criminal Law”, *Current Legal Problems* (2011) 255–286; Hilmi M. Zawati, *Fair Labelling and the Dilemma of Prosecuting Gender-Based Crimes at the International Criminal Tribunals* (Oxford, OUP: 2014).

18 A good review of how these labels were created and recognized at Nuremberg is provided by Ana Filipa Vrdoljak, 'Human Rights and Genocide: The Work of Lauterpacht and Lemkin in Modern International Law', 20 *European Journal of International Law* (2010) 1163–1194; see also Phillip Sands, *East-West Street* (London, Orion Publishing Group: 2017).

19 Dias, (n. 2), 789.

20 Alexander Zahar and Göran Sluiter, *International Criminal Law* (Oxford, OUP: 2008) p. 274; Kai Ambos, *Treatise on International Criminal Law*, 2^nd^ Edition, (Oxford, OUP: 2021).

21 Nersessian, *supra,* (n. 17) 264. The fact that Allied crimes escaped investigation and enquiry altogether is a fair point but tangential to my argument.

22 Makau Mutua goes so far as to say that the London Agreement is the “birth certificate” of the human rights movement, see Makau Mutua, “Savages, Victims, and Saviors: The Metaphor of Human Rights”, *Harvard International Law Journal* (2001) 201–245, 211; Agreement for the Prosecution and Punishment of the Major War Criminals of the European Axis, 8 August 1945, 82 UNTC 280.

23 Even new developments towards the possible establishment of regional criminal courts are founded on the idea of complementarity, see Draft Statute of the Transnational Criminal Court for Latin America and the Caribbean, (“COPLA Draft Statute”), Article 1(2), Article 10, available at: https://en.coalicioncopla.org/documentos (in Spanish). On complementarity see Sarah M.H. Nouwen, *Complementarity in the Line of Fire* (Cambridge, CUP: 2013); Phil Clark, *Distant Justice* (Cambridge, CUP: 2018); see also Neil Boister, 'International Tribunals for Transnational Crimes: Towards a Transnational Criminal Court?', 23 *Criminal Law Forum* (2012) 295–318; Robert J. Currie and Jacob Leon, 'COPLA: A Transnational Criminal Court for Latin America and the Caribbean', 88 *Nordic Journal of International Law* (2019) 587–613. On regional criminal justice developments in Africa see Charles Chernor Jalloh, 'The Nature of the Crimes in the African Criminal Court', 15 *Journal of International Criminal Justice* (2017) 799–826.

24 For example, in the context of western policies of migration control, see Ioannis Kalpouzos and Itamar Mann, “Banal Crimes against Humanity: The Case of Asylum Seekers in Greece”, *Melbourne Journal of International Law* (2015) 1; see also Ioannis Kalpouzos, “International Criminal Law and the Violence against Migrants”, 21 *German Law Journal* (2020) 179.

25 Cryer *et al.*, (n. 15) p. 73.

26 More nuanced rationales in favour of the regional courts also make reference to the dismantling of criminal networks, COPLA Draft Statute, (n. 23) Article 5.

27 Darryl Robinson, “Mexico: The War on Drugs and the Boundaries of Crimes Against Humanity”, 26 May 2015, *EJIL:Talk!,* available online at: https://www.ejiltalk.org/mexico-the-war-on-drugs-and-the-boundaries-of-crimes-against-humanity/ (last accessed 11 November 2020).

28 For a sanguine view of the expansion of U.S. extraterritorial criminal jurisdiction over transnational crimes, see Michael Fabiarz, “Extraterritorial Criminal Jurisdiction” *Michigan Law Review* (2016) 507–557.

29 “Un juez dicta formal prisión a Miguel Ángel Treviño Morales”, el “Z-40”, *Expansión*, 25/07/2013 (available online: https://expansion.mx/nacional/2013/07/25/un-juez-dicta-formal-prision-a-miguel-angel-trevino-morales-el-z-40).

30 U.S. Department of State, Narcotics Rewards Program Targets, available online: https://www.state.gov/narcotics-rewards-program-target-information-brought-to-justice/miguel-angel-trevino-morales-captured/ (last accessed 13/03/2021).

31 Rubén Mosso, “Dan 18 años de cárcel a líder de Los Zetas, responsible de incendio en Casino Royale”, *Milenio*, 21/07/2019, available online at https://www.milenio.com/policia/Zetas-18-anos-lider-responsable-incendio-casino-royale-monterrey (last accessed 24/03/2021).

32 “En Texas, condenan a cadena perpetua a ex líder de Los Zetas”, *Aristegui Noticias*, https://aristeguinoticias.com/0811/Mexico/en-texas-condenan-a-cadena-perpetua-a-ex-lider-de-los-Zetas/ (last accessed 24/03/2021).

33 U.S. Department of Justice, “Former Mexican governor sent to U.S. prison for money-laundering”, *Press Release,* 23/06/2021, available online: https://www.justice.gov/usao-sdtx/pr/former-mexican-governor-sent-us-prison-money-laundering (last accessed 20/07/2021).

34 Allegations that Mexico’s “crackdown” on the cartels during the Calderón administration (2006–2012) was actually only a crackdown on the enemies of the Sinaloa Cartel have been raised by Mexican investigative journalists, see generally Anabel Hernández, *Los Señores del Narco,* (Penguin, Random House: 2010), available in English as *Narcoland – The Mexican Drug Lords and their Godfathers* (Verso, London: 2014); see also Ryan Deveraux, “Prosecution of Top Mexican Security Official Exposes The Façade of the Drug War”, *The Intercept*, 26/01/2020, available at: https://theintercept.com/2020/01/26/mexico-drug-war-el-chapo-garcia-luna-trial/.

35 Indeed, in some instances, this a legal obligation that emerges from the principle of sovereignty.

36 For a view that this “direct criminalization theory” is an exercise in naturalism, see Kevin Jon Heller, “What is an International Crime? (A Revisionist History) *Harvard International Law Journal* (2017) 353–420; on the concept of transnational criminal law see Neil Boister, “Transnational Criminal Law”, *European Journal of International Law* (2003) 953–976; see also Douglas Guilfoyle, “Transnational Crimes” (n. 6).

37 This article focuses on Mexico, as the territorial State where most of the worst violence occurs, and as a State party to the Rome Statute.

38 See comments submitted by Mexico to the International Law Commission in relation to its work on a Draft Convention on Crimes Against Humanity, available online at: https://legal.un.org/ilc/sessions/69/pdfs/spanish/cah_México.pdf at §2 (in Spanish).

39 I am grateful to Sarah Nouwen for helping me to see that point more clearly.

40 Mahmoud Cherif Bassiouni, *Crimes against Humanity in International Criminal* Law (Martinus Nijhoff Publishers, Dordrecht: 1992) and *Crimes against Humanity: Historical Evolution and Contemporary Application* (Cambridge, CUP: 2011); Darryl Robinson, ‘Defining “Crimes Against Humanity” at the Rome Conference’, 93 *AJIL* (1999) 43–57; William Schabas, *The International Criminal Court – A Commentary on the Rome Statute* (Oxford, OUP: 2010) p. 137; Charles Jalloh, “What Makes a Crime Against Humanity a Crime Against Humanity?”, 28 *Am. U. Int’ L. Rev*. (2013) 381–441; Leila Sadat, “Crimes Against Humanity in the Modern Age”, *AJIL* (2013) 334–377; Roger O’Keefe, *International Criminal Law* (Oxford, OUP: 2015) p. 137; Cryer *et al*, *supra,* (n. 15) p. 227; Kai Ambos, *Rome Statute of the International Criminal Court - Article-by-Article Commentary*, 4th Edition, (2022), Article 7.

41 Article 3 ICTRS; Article 7(1) ICCS; *Prosecutor v. Tadić*, ICTY, Trial Chamber, 07/05/1997, §644; *Prosecutor v. Tadić*, ICTY Appeals Chamber, 15/07/1999, §248.

42 Cryer *et al*, *supra*, (n. 15) p. 227; *Tadić*, *ibid*, Trial Chamber Judgment, §625. Therefore, unlike domestic crimes, international crimes raise the possibility that other States will assert prescriptive (universal) jurisdiction over the relevant conduct.

43 Save for the need for a discriminatory intent in relation to persecution as crimes against humanity.

44 Alejandro Rodiles, ‘Law and Violence in the Global South: The Legal Framing of Mexico’s “NARCO WAR”’, *Journal of Conflict and Security Law* (2018) 269–281.

45 Robinson, *supra*, (n. 40) 43–57.

46 Article 7(2)(a), ICCS.

47 Cryer *et al, supra,* (n. 15), p. 227.

48 Guénaël Mettraux, “Crimes against Humanity in the Jurisprudence of the International Criminal Tribunals for the Former Yugoslavia and for Rwanda” *Harvard International Law Journal* (2002) 237–316, 270 and ‘The Definition of Crimes Against Humanity and the Question of a “Policy Element”’, in Leila Sadat, *Forging a Convention on Crimes against Humanity,* (Cambridge, CUP: 2011) pp. 142–176.

49 Mettraux, “Crimes against Humanity in the Jurisprudence of the International Criminal Tribunals for the Former Yugoslavia and for Rwanda”, *ibid*., p. 270.

50 See Article 6(c) Nuremberg Charter, Article 5 ICTYS, Article 3 ICTRS.

51 Sadat, *supra*, (n. 40), 353.

52 *ibid*., 354.

53 *Prosecutor v. Kunarac et al*, Case IT-96-23 & IT-96-23/1-A, Appeals Chamber, Judgment, 12/06/2002, §98.

54 William Schabas, “State Policy as an Element of International Crimes” 98 *Journal of International Criminology* (2008) 953–982, 960.

55 Schabas, *supra*, (n. 40) p. 137; UN Doc. A/CONF.183/C.1/SR.36, para. 13 and UN Doc. A/CONF.183/C.1/SR.34, para. 15.

56 Göran Sluiter, ‘“*Chapeau* Elements” of Crimes Against Humanity in the Jurisprudence of the UN Ad-Hoc Tribunals”, in Sadat (ed.), *supra,* (n. 48). 102–41.

57 It would, therefore, be preferable for Mexico to amend its legislation accordingly.

58 Cryer *et al*, *supra*, (n. 15) p. 235.

59 *ibid*., 236.

60 *Tadić, supra,* (n. 41), Trial Chamber Judgment, §653.

61 *ibid*.

62 Sergio Aguayo and Jacobo Dayán, *El Yugo Zeta – Norte de Coahuila, 2010–2011* (Working Paper: Violence and Peace Seminar, Colegio de México: 2017) p. 7. I will use my own translations from the original Spanish.

63 *ibid*., 7.

64 *ibid*.

65 WOLA Statement, “A Decade After San Fernando Massacre, Migrants Still Face Violence, Impunity for Abuses in Mexico”, 20/08/2020, available online: https://www.wola.org/2020/08/justice-massacre-san-fernando-Mexico-migrants/ (last accessed 23/03/2021).

66 Comisión Nacional de Derechos Humanos, *Informe especial sobre los casos de secuestro contra migantes*, 15/06/2009, at 10. The report is available online at: https://www.cndh.org.mx/sites/all/doc/Informes/Especiales/2009_migra.pdf (last accessed 11/11/2020).

67 Inter-American Commission on Human Rights, *Human Rights of Migrants and Other Persons in the Context of Human Mobility in Mexico*, OEA/Ser.L/V/O, Doc. 48/13, 30/12/2013, §114ff.

68 *ibid*, attributed to Father Pedro Pantoja, General Advisor to domestic civil society organisations.

69 UN, Committee on the Protection of the Rights of All Migrant Workers and Members of their Families, Concluding Observations to Mexico, 03/05/2011, §29.

70 Frédéric Mégret, “Is the ICC Focusing Too Much on Non-State Actors?” in Margaret de Guzman and Diane M. Amann, *Arcs of Global Justice: Essays in Honour of William A. Schabas*, (Oxford, OUP: 2018) pp. 173–201, at 198.

71 Pre-Trial Chamber I, *Prosecutor v Katanga*, ICC-01/04-01/07, 30/09/2008, §396; Pre-Trial Chamber II, *Decision Pursuant to Article 61(7)(a) and (b) of the Rome Statute on the Charges of the Prosecutor against Jean-Pierre Bemba Gombo*, ICC-01/05-01/08-424, 15/06/2009, §81; *Prosecutor v Blaškić*, Case No. IT-95-14-T, ICTY, Judgment, 03/03/2000, §204.

72 Romaine Le Cour Grandmaison, “Order, Sovereignty, and Violence in Mexico”, Mexico Violence Resource Project, 07/12/2020, available online at: https://www.mexicoviolence.org/post/order-sovereignty-and-violence-in-mexico (last accessed 20/07/2021); Ioan Grillo, “How the Sinaloa Cartel Bested the Mexican Army”, *Time*, 18/10/2019, https://time.com/5705358/sinaloa-cartel-Mexico-culiacan/.

73 *Situation in the Republic of Kenya,* Decision Pursuant to Article 15 of the Rome Statute on the Authorisation of an Investigation in the Situation in the Republic of Kenya, ICC-01/19, 31/03/2010, §95.

74 *ibid*., §93.

75 Ginger Thompson, “How the U.S. Triggered a Massacre in Mexico”, *ProPublica*, 12/06/2017. For background to Thompson’s research see, Alice Driver, “How One Reporter Uncovered the US Role in Mexico Massacre”, Global Investigative Journalism Network, 18/06/2018, available online at: https://gijn.org/2018/06/18/how-one-reporter-uncovered-the-us-role-in-a-Mexico-massacre/ (last accessed 13/03/2021); see also Sarah Chayes, *Thieves of State – Why Corruption Threatens Global Security* (New York, Norton: 2016), p. 185.

76 Aguayo and Dayán report that there were approximately 1,450 emergency calls from Piedras Negras and Allende, *supra*, (n. 62) p. 62.

77 Ginger Thompson, *supra,* (n. 75).

78 *ibid*.

79 *ibid*.

80 *Prosecutor v. Kayishema*, ICTR-95-1-T, Judgment, 21/05/1999, §122.

81 *Prosecutor v Limaj* (IT-03-66-T) Judgment, 30/11/2005, para. 182.

82 *Tadić*, *supra*, (n. 41), Trial Chamber Judgment, §649; *Prosecutor v. Kunarac et al*, Case IT-96-23-T & IT96-23/1-T, Trial Chamber, Judgment, 22/02/2001, §415.

83 *Prosecutor v. Akayesu*, Case No. ICTR-96-4-T, 2 September 1998, §581.

84 Although, some of this was paid labour (i.e., working in the “kitchens” drew a salary of $300 per night). Those inmates in maximum security/special isolation were required to dispose of the remains around the prison grounds, and there is no indication this was paid.

85 *ibid*.

86 ICTR Statute, Art. 3; *Tadić*, *supra*, (n. 41), Trial Chamber Judgment, §656; *Akayesu*, *supra* (n. 83), §579.

87 *Tadić*, *supra*, (n. 41), Appeals Chamber Judgment, §248.

88 *Tadić*, (n. 41), Trial Chamber Judgment, §206.

89 *Akayesu*, *supra* (n. 83) §580.

90 *Katanga et al*., *supra*, (n. 71) paras. 394–398; *Bemba*, *supra*, (n. 71), para. 83; *Kenya Decision, supra* (n. 73), §95.

91 *Kunarac et al*, *supra*, (n. 82), §428.

92 Suggesting reflexivity in the context of general principles of liability in international criminal law see Robert Cryer, “Imputation and Complicity in Common Law States – A (Partial) View from England and Wales”, *Journal of International Criminal Justice* (2014) 267–282, 281: “…the point is to develop, reflexively, *principles and rules* from which there may be (again reflexive) deduction in the future [emphasis added]”.

93 Charles C. Jalloh, “What Makes a Crime Against Humanity a Crime Against Humanity?”, 28 *Am. U. Int’ L. Rev.* (2013) 381–441, 435.

94 For context, there are now 90,000 disappeared in Mexico, Sandra Weiss, “México: Más de 90,000 desaparecidos y los crímenes continúan” *DW*, 3 August 2021 available online at: https://www.dw.com/es/m%C3%A9xico-m%C3%A1s-de-90000-desaparecidos-y-los-cr%C3%ADmenes-contin%C3%BAan/a-59031428.

95 *Akayesu*, *supra* (n. 81) §580.

96 *Kunarac et al*, *supra*, (n. 53), §94.

97 *Katanga et al*., *supra*, (n. 71), paras. 397–398.

98 *ibid*.

99 Article 5, ICTYS; Article 3, ICTRS; Article 2, SCSLS; Article 5, ECCCS.

100 *Kunarac et al*, *supra*, (n. 82), §423.

101 Leila Sadat, “Putting Peacetime First: Crimes against Humanity and the Civilian Population Requirement”, *Emory International Law Review* (2017) 197–270; Kai Ambos, ‘The ECCS’s Contribution to Substantive ICL – The Notion of “Civilian Population” in the Context of Crimes Against Humanity’, *JICJ* (2020) 689–700, 690.

102 Ambos, *ibid*., 691.

103 *Tadić*, *supra*, (n. 41), Trial Chamber Judgment, §644.

104 *Kunarac et al*, *supra*, (n. 53), §90.

105 Jacobo Dayán, “Se superó la cifra de 80 mil desaparecidos”, *Aristegui Noticias*, 13/01/2021; Luis Astorga, “The Limits of anti-drug policy in Mexico”, (UNESCO: 2001) pp. 427–431.

106 This approach has been followed at the ICC, see *Prosecutor v Jean Pierre Bemba Gombo* Case No. ICC-01/05-01/08, *Judgment pursuant to Article 74 of the Statute*, 21/03/2016, §153.

107 *ibid*.

108 *ibid.,* §154; Draft Articles on Prevention and Punishment of Crimes Against Humanity, International Law Commission, A/74/10 at 13.

109 ILC Draft Articles, *ibid*, at 36.

110 *Kunarac et al*, *supra*, (n. 53), §91–92.

111 *ibid*., §91.

112 *Bemba Gombo*, *supra*, (n. 71), §674.

113 *ibid*., §153.

114 Three “communications” pursuant to Article 15 Rome Statute have been sent to the Office of the Prosecutor (OTP): *Mexico: Report on the Alleged Commission of Crimes Against Humanity in Baja California between 2006–2012* (2014) available online at: https://www.fidh.org/IMG/pdf/mexique642ang2014web.pdf (this petition was rejected); *Mexico Coahuila: Ongoing Crimes Against Humanity* (2017) available online at: https://www.fidh.org/IMG/pdf/angmexico_coahuila_ongoing_crimes_against_humanity_fidh-final_a_revisar-1.pdf; *Article 15 Communication Under the Rome Statute to the International Criminal Court Regarding the alleged commission of crimes against humanity in Chihuahua, México between 2008 and 2010* (2018) https://www.fidh.org/IMG/pdf/mexique715anglais-1_final.pdf (all the reports last accessed on 11 November 2020). Of these communications, only the first has received a response from the OTP (rejected).

115 Aguayo and Dayán, *supra*, (n. 62); *Coahuila: Ongoing Crimes Against Humanity* (2017) available online at:https://www.fidh.org/IMG/pdf/angMexico_coahuila_ongoing_crimes_against_humanity_fidh-final_a_revisar-1.pdf.

116 *Article 15 Communication Under the Rome Statute to the International Criminal Court Regarding the alleged commission of crimes against humanity in Chihuahua, México between 2008 and 2010* (2018) https://www.fidh.org/IMG/pdf/mexique715anglais-1_final.pdf (all the reports last accessed on 11 November 2020). On selectivity see Robert Cryer, *Prosecuting International Crimes: Selectivity and the International Criminal Law Regime* (Cambridge, CUP: 2005); J. Eskauriatza, ‘The “Cienfuegos Affair” and the Proposal for a New Regional Transnational Criminal Court in Latin America (COPLA)’, *OpinioJuris*, 16 July 2021, available online at: http://opiniojuris.org/2021/07/16/the-cienfuegos-affair-and-the-proposal-for-a-new-regional-transnational-criminal-court-in-latin-america-copla/ (last accessed 11 August 2021).

117 Beverley Goldberg, “Ayotzinapa: 1,826 days later, and still no justice”, *Open Democracy*, 30 September 2019, available online: https://www.opendemocracy.net/en/democraciaabierta/masacre-de-ayotzinapa-5-a%C3%B1os-sin-resolver-en/ (last accessed 21 September 2021). For a reconstruction of the events that occurred on the 26th September 2014 (based on: i) eyewitness accounts, ii) video-footage, iii) testimony of the surviving student Fernando Marín, iv) information from Tlachinollan Human Rights Centre) see Anabel Hernández, “The Hours of Extermination – The Disappearance of the Forty-Three Students in Iguala, Guerrero”, in Lydia Cacho, *et al* (n. 5) at 102–120.

118 Sarah Chayes, *Thieves of State – Why Corruption Threatens Global Security* (New York, Norton: 2016), 185.

119 Naomi Roht-Arriaza and Santiago Martínez, ‘Grand Corruption and the International Criminal Court in the “Venezuela Situation”’, *Journal of International Criminal Justice* (2019) 1057–1082, 1062.

120 Decision on the Prosecutor’s request for authorization of an investigation pursuant to Article 15(3) of the Statute, Situation in the Republic of the Philippines, Pre-Trial Chamber I, 15 September 2021, ICC-01-21, available online from the Court’s website.

121 Martti Koskenniemi, *The Gentle Civilizer of Nations: The Rise and Fall of International Law 1870–1960* (Cambridge: Cambridge University Press: 2001), 489.

122 Anthony Anghie, *Imperialism, Sovereignty and International Law*, (New York, CUP: 2004).

123 Dias, (n. 2) 790, referring to the International Convention on Civil and Political Rights (“ICCPR”), Article 14(3)(a).

124 Mexico acceded to the ICCPR on 23 March 1981.

125 John Gardner, “Legal Positivism - 5 1/2 Myths”, 46 *American Journal of Jurisprudence* 1 (2001) 199, 200.

126 For a comprehensive list see Dias, (n. 2) at fn. 23, 793.

127 Immi Tallgren, 'The Sensibility and Sense of International Criminal Law' 13 *European Journal of International Law* (2002) 561–595; Carsten Stahn, *A Critical Introduction to International Criminal Law* (Cambridge, CUP: 2019), 117–158; Guilfoyle, (n. 17) at 257, citing Gerry Simpson, “Men and Abstract Entities: Individual Responsibility and Collective Guilt in International Criminal Law” in André Nollkaemper and Herman van der Wilt, *System Criminality in International Law* (Cambridge, CUP: 2009); see also Janine Natalya Clark, 'The Limits of Retributive Justice: Findings of an Empirical Study in Bosnia and Hercegovina' 7 *Journal of International Criminal Justice* (2009) 463–487.

128 Koskenniemi, (n. 13) 14.

129 Mark Drumbl, *Atrocity, Punishment and International Law* (Cambridge, CUP: 2009).

130 See *Propuesta Ciudadana Para La Construcción de Una Política Sobre Verdad, Justicia y Reparación a las Víctimas de la Violencia y de las Violaciones a Derechos Humanos* (2019), (“Citizens Proposal”); see also https://jtMexico.org/; On the turn to transformative justice in transitional justice literature see, Kieran McEvoy, “Beyond Legalism: Towards a Thicker Understanding of Transitional Justice”, *Journal of Law and Society* (2007) 411–440; Dustin Sharp, “Interrogating the Peripheries: The Preoccupations of Fourth Generation Transitional Justice”, 26 *Harvard Human Rights Journal* (2013) 149–178; Paul Gready and Simon Robins (eds.) *From Transitional to Transformative Justice* (Cambridge, CUP: 2019).

131 Part 5, “Final Agreement for the End of Conflict and the Construction of a Stable and Long Lasting Peace”, (hereafter the Peace Agreement) signed on 24 November 2016 and ratified by Congress 1 December 2016. The full text of the peace agreement is available at: http://www.altocomisionadoparalapaz.gov.co/procesos-y-conversaciones/Documentos%20compartidos/24-11-2016NuevoAcuerdoFinal.pdf.

132 *ibid*.

133 See Christopher Paul, Colin P. Clarke and Chad C. Serena, *Mexico is Not Colombia*, (Rand Corporation: 2014) xvii. Even so, the authors identify the following cases as the most analogous to Mexico’s situation: Colombia, Peru, the Balkans (especially Bosnia and Croatia), West Africa (especially Sierra Leone and Liberia) and the Caucasus (Georgia and Chechnya). Other cases that made the shortlist are Angola, Myanmar, Tajikistan and Afghanistan.

134 I do not assert that the “war on drugs” began in 2006.

135 See for example, *Article 15 Communication Under the Rome Statute to the International Criminal Court Regarding the alleged commission of crimes against humanity in Chihuahua, México between 2008 and 2010* (2018) https://www.fidh.org/IMG/pdf/mexique715anglais-1_final.pdf (last accessed 11 October 2020). See also the Report of the Special Rapporteur on torture and other cruel, inhuman or degrading treatment or punishment, Juan E. Méndez, 29 December 2014, A/HRC/28/68/Add.3, available online at: http://antitorture.org/mexico-2014/ (last accessed 11 November 2020) and United Nations Convention against Torture and Other Cruel, Inhuman and Degrading Treatment or Punishment, Concluding Observations on the seventh periodic report submitted of Mexico, CAT/C/MEX/CO/7, 24^th^ July 2019, at §8, available online at: https://tbinternet.ohchr.org/_layouts/15/treatybodyexternal/Download.aspx?symbolno=CAT/C/MEX/CO/7&Lang=En (last accessed 11 November 2020).

136 Linnea Sandin, “Femicides in Mexico: Impunity and Protests”, Centre for Strategic & International Studies, 19^th^ March 2020, available online at: https://www.csis.org/analysis/femicides-mexico-impunity-and-protests (last accessed 11 November 2020).

137 Throughout 2018, 2019, and 2020, the murder rate has remained steady at 29 homicides per 100,000 https://politica.expansion.mx/mexico/2021/07/27/homicidios-en-mexico-2020-inegi (last accessed 22 September 2021). It amounts to about 36,000 official murders a year.

138 “El tamaño de la impunidad en México”, *Impunidad Cero*, available online: https://www.impunidadcero.org/impunidad-en-mexico/#/ (last accessed 13 October 2021); Alejandro Anaya-Muñoz, James Cavallaro, and Patricia Cruz-Marín, *La impunidad active en México: Cómo entender y enfrentar las violaciones masivas a los derechos humanos*, ITESO-Universidad Jesuita de Guadalajara and University Network for Human Rights (ITESO, Guadalajara: 2021).

139 The slogan rhymes in Spanish: *abrazos no balazos*. See National Development Plan and National Security and Peace Plan, Spanish language versions available online: National Development Plan https://www.gob.mx/cenace/acciones-y-programas/plan-nacional-de-desarrollo-2019-2024-195029 and National Peace and Security Plan: https://lopezobrador.org.mx/wp-content/uploads/2018/11/PLAN-DE-PAZ-Y-SEGURIDAD_ANEXO.pdf (each last accessed 25/03/2021).

140 AMLO speech on 30/01/2019, as reported by Hannia Novell, “AMLO: el fin de la guerra contra el narco y su amnistía”, *Eje Central*, 07/02/2019, available online: http://www.ejecentral.com.mx/bitacora-de-guerra-amlo-el-fin-de-la-guerra-contra-el-narco-y-su-amnistia/. See also, Arturo de Dios Palma, “AMLO analiza amnistía a líderes del narco para garantizar la paz, *El Universal*, 02/12/2017, available online: https://www.eluniversal.com.mx/elecciones-2018/amlo-plantea-analizar-amnistia-lideres-del-narco-para-garantizar-la-paz and David Agren, “Fury as Mexico presidential candidate pitches amnesty for drug cartel kingpins”, *The Guardian*, 04/12/2017, available online: https://www.theguardian.com/world/2017/dec/04/mexican-presidential-candidate-pitches-amnesty-deal-for-drug-cartel-kingpins (all last accessed 06/09/2019). The translations from the original Spanish are my own.

141 Morning Press Conference, 30/01/2019, transcript available online: https://www.gob.mx/presidencia/prensa/conferencia-de-prensa-del-presidente-andres-manuel-lopez-obrador-30-de-enero-de-2019 (last accessed 11/11/2020).

142 “Mexican President authorizes 4 more years of military policing”, *CBS News*, 21/05/2020, https://www.cbsnews.com/news/mexico-military-authorized-president-lopez-obrador-4-more-years-domestic-policing-cartel-crime/#:~:text=Mexican%20president%20authorizes%204%20more%20years%20of%20military%20policing&text=Mexico%20City%20%E2%80%94%20Mexico's%20president%20published,more%20years%2C%20to%20March%202024.

143 Juan Rulfo, “Nos han dado la tierra”, in *Pedro Páramo y el Llano en Llamas* (Planeta, Barcelona: 2006) 137. One possible English translation would be: “We do not say what we think. It has been a long time since we felt the desire to say anything.”

144 Sander Wirken and Hanna Bosdriesz, “Privatization and Increasing Complexity of Mass Violence in Mexico and Central America: Exploring Appropriate International Responses” in Harmen Van der Wilt and Christophe Paulussen (eds), *Legal Responses to Transnational and International Crimes – Towards an Integrative Approach* (Edward Elgar Publishing, Cheltenham: 2017) 245–251.

145 *ibid*.

146 The first being the War of Independence from Spain (1810–1821), the second refers to the Benito Juárez Reform Law Period (1858–1861) and, thirdly, the Revolutionary uprising, (1910–1917).

147 Whether this meeting led to the capture of El Chapo, as the Mexican authorities have claimed, is disputed, see Benjamin Lee and Scott Bixsby, ‘Sean Penn on El Chapo interview: “I have a terrible regret”’, 15 Jan 2016, *The Guardian*, available online at: https://www.theguardian.com/film/2016/jan/15/sean-penn-on-el-chapo-interview-i-have-a-terrible-regret (last accessed on 11 November 2020).

148 Howard Campbell and Tobin Hansen, “Getting out of the Game: Desistance from Drug-Trafficking”, *International Journal of Drug Policy*, (2012) 481–487, 486.

149 Ruti Teitel, *Transitional Justice* (Oxford, OUP: 2000) 27–67.

150 Gerry Simpson, “International Criminal Law – The Next Hundred Years”, in Heller *et al*, (n. 5) 841–849, 847.

151 *ibid*., 848.

152 Robert Cryer, “The Role of International Criminal Prosecutions in Increasing Compliance with International Humanitarian Law in Contemporary African Conflicts” in Heike Krieger, *Inducing Compliance with International Humanitarian Law: Lessons from the African Great Lakes Region* (Cambridge, CUP: 2015) 188–216; Jakob von Holderstein Holstermann, ‘A “Slice of Cheese” – a Deterrence-Based Argument for the International Criminal Court’, 11 *Human Rights Review* 289–315.

